# FACTORS ASSOCIATED WITH POOR SELF-RATED HEALTH BETWEEN AGES 70 AND 95: THE JERUSALEM LONGITUDINAL AGING STUDY

**DOI:** 10.1093/geroni/igae098.2401

**Published:** 2024-12-31

**Authors:** Irit Stessman-Lande, Aliza Rozenberg, Jeremy Jacobs, Jochanan Stessman

**Affiliations:** Hebrew University of Jerusalem and Hadassah Medical Center, Jerusalem, Yerushalayim, Israel; Hebrew University of Jerusalem and Hadassah Medical Center, Jerusalem, Yerushalayim, Israel; Hebrew University of Jerusalem/Hadassah Medical Center, Mount Scopus, Jerusalem, Yerushalayim, Israel; Hebrew University of Jerusalem and Hadassah Medical Center, Jerusalem, Yerushalayim, Israel

## Abstract

Self-rated health assessment is widely used among individuals aged over 70 to subjectively gauge health status, with poor self-rated health associated with shorter life expectancy. This study aimed to identify risk factors for poor self-rated health between ages 70-95. The Jerusalem Longitudinal Aging Study (1990-2023) prospectively follows a representative community-dwelling cohort born 1920-21, assessed during home-visit during 1990, 1998, 2005, 2010, 2015 at ages 70, 78, 85, 90, and 95 (n=604, 1024, 1222, 729, 508) respectively. Participants answered the question “Do you feel healthy?”; those answering “no” were defined as having poor self-rated health. Depression emerged as the most significant risk factor for declining self-rated health, with a 3-4.6 times higher risk compared to those without depression across different age groups (P≤0.044). Self-reported financial difficulty, physical inactivity, impairments in activities of daily living, and background comorbidity showed inconsistent correlations with declining self-rated health. There was a robust statistically significant association between self-rated health and mortality across all ages between 70-100 years old, with poor self-rated health consistently predicting shorter life expectancy. Even after adjusting for other risk factors, the association between poor self-rated health and mortality remained significant. The findings suggest that while commonly accepted risk factors for illness might not directly correlate with decline in self-rated health, nonetheless depression emerges as a significant predictor. This implies that poor self-rated health could be a symptom of underlying affective status. Overall, the study underscores the importance of considering mental health factors in assessing self-rated health and predicting longevity between ages 70-100.

